# Machine vision-based recognition of safety signs in work environments

**DOI:** 10.3389/fpubh.2024.1431757

**Published:** 2024-11-27

**Authors:** Jesús-Ángel Román-Gallego, María-Luisa Pérez-Delgado, Miguel A. Conde, Marcos Luengo Viñuela

**Affiliations:** Escuela Politécnica Superior de Zamora, Universidad de Salamanca, Avda, Requejo, Zamora, Spain

**Keywords:** occupational risk, prevention, convolutional neural networks, image recognition, classification

## Abstract

The field of image recognition is extensively researched, with applications addressing numerous challenges posed by the scientific community. Notably among these challenges are those related to individual safety. This article presents a system designed for the application of image recognition in the realm of Occupational Risk Prevention—a concern of paramount importance due to the imperative of preventing workplace accidents as falls, collisions, or other types of accidents for the benefit of both workers and enterprises. In this study, convolutional neural networks are employed due to their exceptional efficacy in image recognition. Leveraging this technology, the focus is on the recognition of safety signs used in Occupational Risk Prevention. The primary objective is to enable the recognition of these signs regardless of their orientation or potential degradation, phenomena commonly observed due to regular exposure to environmental elements or deliberate defacement. The results of this research substantiate the feasibility of integrating this technology into devices capable of promptly alerting individuals to potential risks. However, to improve classification capabilities, especially for highly degraded or complex images, a larger and more diverse data set might be needed, including real-world images that introduce greater entropy and variability. Implementing such a system would provide workers and companies with a proactive measure against workplace accidents, thereby enhancing overall safety in occupational environments.

## 1 Introduction

In the event of a workplace accident befalling a worker, it poses a multifaceted challenge for both the company and its production system, however, if hazards could be avoided through effective signaling and recognition, many issues for both workers and the company could be significantly reduced. Occupational Risk Prevention (ORP) can be precisely delineated as a comprehensive array of activities or measures meticulously formulated to preempt or alleviate potential risks inherent within a workplace milieu, and as expected, the signaling related to specific risks inherent to the job is encompassed within these activities. Within this conceptual framework, an occupational risk manifests as the plausible occurrence of a work-related accident leading to injuries, illnesses, and fatalities.

The manifestation of accidents not only exerts deleterious impacts on the economic facets of the company but also impinges upon human resources (1). Signage assumes a pivotal role within the context of ORP, serving as a critical mechanism to apprise workers of specific hazards intrinsic to their workplace surroundings. Consequently, it becomes indispensable to impart targeted training to workers for the adept understanding and interpretation of these visual cues. Numerous scientific investigations have been conducted to explore the comprehension of safety signs across diverse industrial sectors (2–6). A consistent finding across these studies is the limited understanding of safety pictograms. The determinants contributing to this outcome can be categorized into two primary groups: human factors and pictogram attributes. The age and experience of the worker are significantly influenced by these factors. Conversely, inadequate training or the presence of distractions that hinder sign observation diminishes the effectiveness of safety signs (6). Regarding pictogram features, key considerations include the visibility of the safety sign, image quality, and the symbols employed. Additionally, it is imperative to acknowledge the potential impairment of safety signs, such as damage, rotation, partial concealment, or distance from the observer’s location. In all these scenarios, training becomes inconsequential, and the risk of occupational accidents significantly escalates. The issues delineated in the preceding paragraph underscore the necessity for a system with the capability to autonomously identify safety signs in real-time. Such a system could effectively alert workers to potential hazards, thereby facilitating the optimization of corporate efforts to mitigate occupational accidents. It is evident that the advantages inherent in any security-enhancing system are substantial across various dimensions (7, 8). The visual sense holds paramount importance in our communication processes. Despite the significance of the four other senses, vision becomes particularly crucial in environments where occupational hazards pose risks to workers. Consequently, images assume a foundational role in the communicative framework of such work environments. The expeditious interpretability of images, as opposed to explanatory text, is highlighted by research indicating that individuals require less time to comprehend visual information (9). Consequently, the response time to visually transmitted information is markedly quicker.

**Figure 1:**
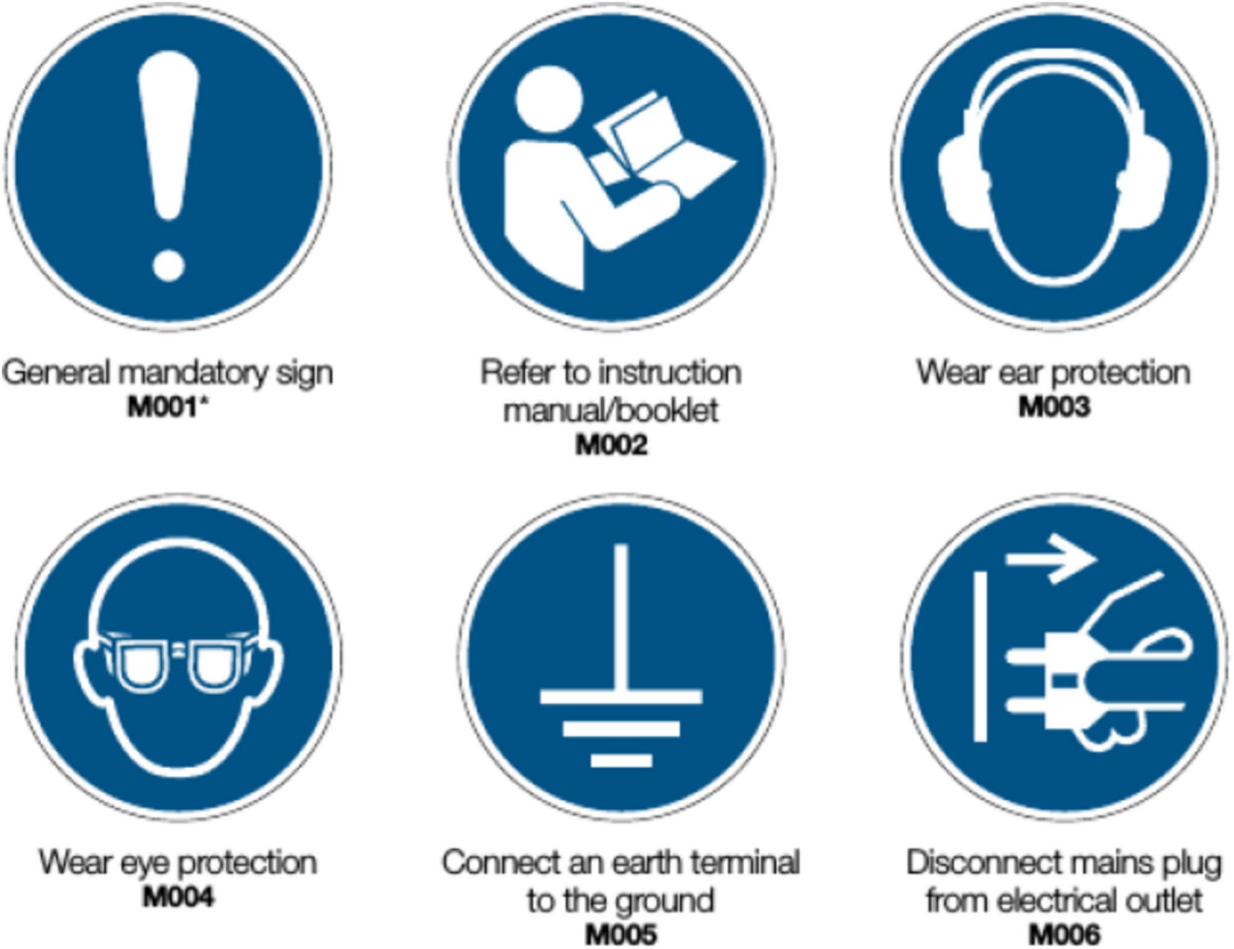
Example of ORP mandatory action signs (48).

In adherence to regulations set forth by the International Organization for Standardization, as articulated in document ISO 17724:2003 (10), safety signs employed in ORP serve the purpose of conveying messages aimed at safeguarding the physical well-being of workers. Furthermore, the pictograms utilized exhibit distinctive shapes and colors that facilitate prompt identification of associated risks, thereby conveying a safety message when the prescribed recommendations accompanying these safety signs are adhered to. The ISO 7010 standard specifies five combinations of shapes and colors within its defined pictograms, each serving to convey information of various types. Figure 1 shows a set of mandatory signs.

1. Safe condition sign: green color and square or rectangular shape.
2. Fire equipment sign: red color and square shape.
3. Mandatory action sign: blue color and circular shape.
4. Prohibition sign: red color and circular shape with a diagonal line.
5. Warning sign: yellow color and equilateral triangle with rounded corners.

Numerous research articles have demonstrated the feasibility of proficiently recognizing signals and subsequently interpreting them for real-time decision-making, as evidenced by existing literature (11–13). Convolutional Neural Networks (CNNs) (14, 15) represent a technology currently deployed in this domain (16–18). The increasing popularity of CNNs in recent years is attributed to their noteworthy performance in addressing challenges within computer vision, a domain previously deemed nearly insurmountable (19, 20). It is noteworthy that CNNs currently stand as one of the foremost methods for image classification (21, 22).

Considering the above, how can machine vision systems be improved for automated recognition of ORP safety signs in industrial environments? This study aims to address the limitations of existing research by focusing on automated recognition of ORP signs using machine vision, a less explored area compared to traffic sign recognition. While most studies focus on improving machine vision algorithms for traffic sign detection, this work aims to fill the existing gap by investigating how to adapt and optimize these systems for working environments, where ORP signs may present variations in design, color and context.

CNNs are a class of deep learning models primarily used for image and video analysis. They are designed to automatically and adaptively learn spatial hierarchies of features through a process of convolution, pooling, and activation. CNNs are particularly effective at tasks like image recognition, object detection, and segmentation due to their ability to capture local patterns and handle spatial data.

A CNN operates as a trainable system, acquiring the ability to solve problems through learning from diverse examples. Consequently, the availability of a sufficiently extensive and representative image dataset is imperative for the effective training of the network. Despite the existence of publicly accessible image datasets for training networks, none, to the best of the authors’ knowledge, are tailored to the specific problem under consideration in this article. Therefore, it becomes imperative to generate a substantial dataset of images tailored to our unique problem to facilitate the application of a CNN.

The primary aim of the research elucidated in this article is the comprehensive analysis of the performance of a CNN specifically crafted for the detection and classification of images depicting safety signs within ORP contexts, given that most of the research in this area focuses on traffic signaling, rather than occupational hazard prevention signaling. Constructing the system mandates the precise definition of the network architecture and the creation of a curated image dataset to train the CNN effectively.

To assemble this dataset, an initial selection of images was made from the principal safety signs employed in ORP. Subsequently, the dataset was augmented by incorporating additional images generated through transformations applied to the original set. These transformations aim to replicate conceivable imperfections exhibited by signs in real-world scenarios, as well as challenges associated with their display. The Supplementary material associated with this article includes additional information on the data augmentation process. Following the training phase with this augmented dataset, the network’s proficiency in recognizing novel images was analyzed.

It is necessary to note that the closest comparable work addressing related concerns is documented in (23), where a CNN is employed to assess the degradation level of images portraying safety signs. It is crucial to emphasize, however, that the focus of the problem addressed in both articles differs significantly.

The problem of image classification constitutes a foundational challenge within the domain of computer vision. A myriad of methodologies has been employed over the years for this purpose, encompassing nearest neighbor classification, decision trees, fuzzy classifiers, neural networks, and support vector machines (24). Generally, these approaches necessitate a preliminary segmentation step or feature extraction operation prior to the classification process. Contemporary advancements in image classification techniques have yielded methods that exhibit superior performance without the prerequisite of preprocessing steps such as image segmentation or feature extraction. Among these modern approaches, CNNs have garnered considerable acclaim and currently stand as one of the most prevalent methods (22, 25). CNNs possess the unique ability to discern visual patterns directly from raw pixel data, obviating the need for preliminary feature extraction steps inherent in alternative image classification methods. Unlike conventional methods, a CNN inherently learns these features. Notably, the efficacy of CNNs is contingent upon the availability of extensive datasets for training. The proliferation of large-scale public image datasets comprising millions of high-resolution images in recent years has empowered CNNs to achieve remarkable performance in image classification tasks. Prominent examples include the utilization of the CNN architecture known as AlexNet, as demonstrated in the research presented by Krizhevsky et al. (26). Other noteworthy CNN models applied in image classification encompass VGG-16 (27), ResNet (28), and GoogLeNet (29).

Within the realm of ORP safety signs and image classification, extant literature predominantly focuses on human driven visual identification and classification. This body of work explores the classification aptitude of individuals with diverse characteristics, including workers, technicians, and the public (2–6). Recent scientometric analysis covering literature from 1990 to 2019 regarding safety signs reveals two pivotal observations: firstly, safety signs represent an emerging research field in a phase of rapid development; secondly, traffic signs and driving safety predominate as the most prevalent research topics (30). While the exploration of safety signs appears intriguing, research has primarily concentrated on traffic signs. Despite the proliferation of publicly available traffic sign datasets, such as GTSRB (31), BTSD (32), and STS (33), limited attention has been directed towards datasets specific to ORP safety signs. This dearth hampers research endeavors in this area, despite its inherent importance.

Traffic sign identification and classification, necessitating the recognition of objects in outdoor environments, encounter challenges influenced by factors like lighting conditions, sign position and rotation, degradation, and the presence of obstructive objects. Analogous challenges are inherent in the classification of ORP safety signs. Existing methods for traffic sign classification include neural networks, k-nearest neighbor methods, support vector machines, and binary-tree-based classification (34). Typically, these methods leverage color and shape attributes for detection and classification. Research presented in (35) underscores the accuracy of various methods, reporting values ranging between 96.5 and 98.5% in traffic sign classification. Recent years have witnessed a surge in solutions applying CNNs to classify traffic signs (36, 37). This approach circumvents the descriptor extraction step sensitive to various factors in prior methods, allowing CNNs to utilize traffic sign images as input and autonomously learn detailed descriptions. Research in (38) demonstrates the superiority of CNNs (98.3% accuracy) over the random forest method (96.1% accuracy) and comparable performance to human observers (98.8% accuracy). Notably, reported precision levels for CNNs even exceed 99%. Despite the extensive studies on traffic sign classification, research specific to ORP safety signs is sparse. While datasets for traffic signs are abundant, a dedicated dataset for ORP safety signs is conspicuously absent, limiting research prospects in this domain.

As of our current knowledge, the sole research applying CNNs to ORP safety signs is encapsulated in a recent article by Mu and Yue (23). This investigation delves into the impact of image degradation on machine recognition of safety signs. The methodology employs a CNN for image classification and introduces a set of degraded images to train the network. It deviates from our approach in that the CNN’s objective is to identify the applied degradation within the input image. Our study investigates the performance of a CNN for ORP safety signs recognition under various image degradation conditions, with the aim of improving the robustness of this type of systems in real scenarios. The initial dataset comprises 108 images of safety signs conforming to ISO 7010, with 12 types of simulated image degradation applied (blurry, corroded, cracked, dilated, fading, frosted, leaning, shadowed, shaking, shielded, stretched, and wrinkled). The employed CNN architecture features a Convolution layer with 32 convolution kernels, an Activation layer with ReLU function, a Max-pooling layer, and a final Softmax layer with 12 outputs denoting the identified degradation type. Computational results indicate a network accuracy of 98.16% for the training set and approximately 97% for new images. Notably, while numerous studies address traffic sign degradation, the research in (23) encompasses a broader spectrum of degradation types. It is pertinent to acknowledge, however, that certain distortions considered in this research (cracked, dilated, stretched, and wrinkled) may not be practically feasible in real-world pictograms.

## 2 Materials and methods

CNNs represent a distinctive subset within the broader domain of Artificial Neural Networks (ANNs), and their efficacy has been notably demonstrated in tasks such as image recognition and classification (21, 22). The nomenclature “Convolutional” is derived from one of their pivotal hidden layers, known as the Convolutional layer. As alluded to earlier, the widespread adoption of CNNs in recent years is attributed to their commendable performance in addressing challenges within computer vision domains (19, 20).

Convolutional Neural Networks share a common architectural framework with multilayer ANNs, but their input is specifically tailored to accommodate images. Within the CNN, the hidden layers play a pivotal role in discerning distinctive features within images, enabling the network to identify and differentiate them, thereby facilitating the categorization of input images into classes. The functioning of a CNN bears semblance to human vision, where the recognition of an object entails the identification of its constituent elements, irrespective of potential omissions. However, the recognition of individual components alone is insufficient; the spatial arrangement of these components is a critical determinant. Consequently, a CNN is not merely tasked with recognizing an object but must also acquire an understanding of the spatial relationships between its elements, encompassing their relative sizes and colors.

The successive layers within a CNN are responsible for uncovering the features that characterize an image. Each layer applies varying levels of abstraction to detect different features. As the image traverses through the network, these features are constructed hierarchically, yielding a spectrum from low-level abstraction features (e.g., edges) in the initial layers to higher-level abstraction features (e.g., object shapes) in the later layers (14, 15).

### 2.1 Convolutional neural network layers

**Figure 2:**
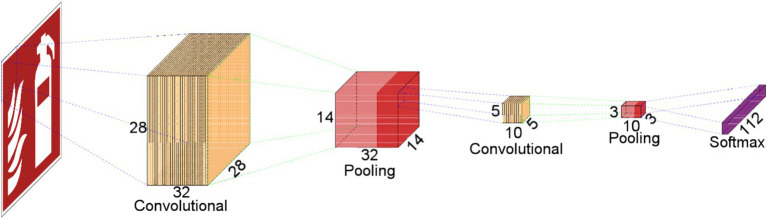
A basic convolutional neural network scheme.

There are three basic layers that define a CNN: *Convolution Layer, Pooling Layer and Softmax Layer*. Each of these layers has been designed to perform a very specific task within the CNN. Figure 2 shows an example of a CNN architecture composed of two Convolution layers, two Pooling layers and a Softmax layer. In the following subsections we will review each of these layers.

#### 2.1.1 Convolutional layer

The Multi-Layer Perceptron (MLP) (39) is a popular ANN made up of multiple densely connected layers that learn global patterns. In contrast, the Convolution layers of a CNN learn local patterns, which means that they learn a pattern at certain coordinates of the image and can find it elsewhere in the image. A Convolutional layer applies *filters* (also called *kernels* or *feature detectors*) to extract features. The output obtained as a result of the convolution of the image and a filter is called *feature map*, *convolved feature* or *convolved map*. The convolution operation captures the local dependencies that exist in the original image. The essential parameters for this layer entail defining the set of learnable filters and specifying their dimensions. Each filter possesses a predetermined width, height, and depth equivalent to that of the input volume.

In the context of image processing, the input volume is defined by the pixels constituting the image. In instances where the RGB color model represents the image, each pixel is characterized by three values denoting the intensity of red, green, and blue. Consequently, the depth of the input volume is three. As the filter traverses the image from the top-left to the bottom-right, the convolution operation is systematically applied to the window’s position. Each instance of this convolution operation yields a value in the feature map. As the filter progresses across the input volume, a two-dimensional activation map emerges, providing the responses of that filter at every spatial position. Thus, the network learns filters that activate when detecting specific visual features represented by said filters. These features manifest as rudimentary entities, such as edges, in the initial layers of the CNN and evolve into more intricate structures, such as complete circumferences, in deeper layers. A filter representing a particular feature informs us about the presence, frequency, and location of that feature within the image.

Application of convolutions to an image result in a reduction in size contingent upon the filter dimensions. To counteract this size reduction, a technique known as *padding* is employed. This method involves incorporating a margin around the input image, ensuring that the feature maps generated by the filter maintain the original image size. While the convolution operation may incorporate a larger step to decrease image size, it is crucial to note that reducing image size need not be detrimental. Indeed, such operations, as will be elucidated in subsequent steps, are integral to the overall process. Alternatively, intermediate rows and columns can be introduced into the image as padding to enlarge it. The impact on the learning algorithm, in terms of computation time, is contingent upon the specific problem under consideration.

When a filter fails to discern any discernible features, signifying the absence of a valid feature, the filter undergoes modification during the backpropagation stage of the network. Backpropagation, a well-established method for weight adjustment in ANNs (40), takes on a distinctive form in CNNs by incorporating convolution into the process (41).

In summary, Convolution layers employ filters on either the primary image or a feature map within a CNN, contingent upon the layer’s position in the CNN architecture. Multiple filters within a Convolution layer are applied to its input to extract diverse features while concurrently learning the distinctive attributes of each filter. Consequently, each filter generates dissimilar feature maps from the same original image.

#### 2.1.2 Pooling layer

In the realm of scientific analysis, this particular stratum is commonly integrated immediately subsequent to a Convolution layer with the intent of diminishing the dimensions of feature maps and consolidating the information transmitted to subsequent layers. Analogous to the Convolution layer, the operations executed within this stratum involve the systematic traversal of a preset-sized window across the received feature. The distinguishing factor lies in the methodology applied to the affected elements and the non-overlapping nature of the window.

Within this context, the window can execute three distinct operations aimed at reducing the dimensions of the image. The min-pooling operation entails extracting the minimum value within the window, while the average-pooling operation compresses information by computing the average of values enclosed within the window. Lastly, the max-pooling operation involves extracting the maximum value found within the window.

#### 2.1.3 Softmax layer

Situated as the concluding stratum within a CNN, this layer holds the pivotal responsibility of ascertaining the class to which the network attributes the presented input image. Comprising an equivalent number of outputs as the distinct images the CNN is capable of recognizing, this layer calculates the probability associated with each specific class, thereby delineating the likelihood that the given image belongs to a particular category.

The input vector and the output vector of this layer are the same size. The elements of the input vector can take any real value, while the output vector includes real values whose sum is 1. Let $\vec{x}=\left(x_{1},..,n_{n}\right)$ denote the input vector of this layer, where *n* is the number of classes. Equation 1 defines the Softmax function applied to the *xi* element of the input vector.


(1) $$S\left(x_{i}\right)=\frac{e^{x_{i}}}{\sum_{j=1}^{n}e^{x_{j}}}$$

The Softmax function applies the exponential function to each element of the input vector, giving larger values more weight. As a result, a value greater than 0 is obtained, which increases as the value of the input increases. The Softmax function exposes the main differences, and this is very useful to speed up and facilitate learning.

Throughout the years, various CNN architectures have been proposed, as documented in the literature (26, 42). Many of these architectures leverage fundamental concepts established by the LeNet architecture (42). In the context of image recognition, the LeNet architecture is employed to categorize input images into predefined classes. The network integrates four key operations for image recognition: convolution, non-linearity, pooling, and classification:

- Convolution: Detects features in the image by applying a filter that highlights edges and patterns.
- Non-linearity: Uses functions like ReLU to capture complex relationships, adding flexibility to the model.
- Pooling: Reduces the image resolution to retain the most important features and simplify the model.
- Classification: Employs final layers to assign probabilities to different classes, identifying the image’s class.

Notably, the non-linearity aspect has not been expounded upon in the preceding discussion. Thus, we will elucidate on it now. During the training phase of the network, the incorporation of a non-linear activation function becomes imperative for capturing intricate relationships within the data. The Rectified Linear function has proven to be the most efficacious non-linear activation function for CNNs. Neurons employing this function are denoted as Rectified Linear Units (ReLUs). The computation of this function is defined by Equation 2, where the max operation determines the maximum value between the two arguments.


(2) $$\mathrm{ReLU}\left(x\right)=\max\left(0,x\right)$$

**Figure 3:**
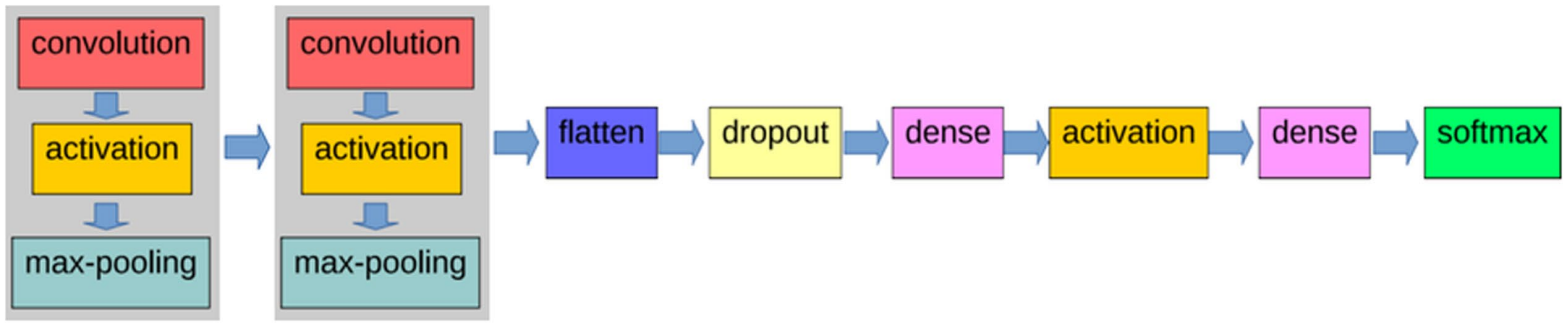
Layers that define the CNN to classify ORP-related images.

CNN devised for the identification of ORP images is constructed based on the fundamental operations inherent in the LeNet architecture. Illustrated in Figure 3, the foundational architecture of the CNN developed in this study for ORP image identification encompasses a total of 12 layers. The initial two blocks are dedicated to feature extraction, while the subsequent layers are employed for classification purposes.

The CNN operates on input images of dimensions 28 × 28 pixels, and it is configured with 112 outputs to facilitate the detection of 112 distinct pictograms. Following the completion of the training phase, the network is poised for pictogram identification. When presented with an image as input, the network activates the output corresponding to the recognized pictogram.

The initial part of the architecture is defined by two groups featuring identical layers. Each group comprises a Convolution layer, an Activation layer utilizing the ReLU function, and a Max-pooling layer. As the filters of the first layer traverse the image, a feature map is generated for each filter. Subsequently, the ReLU function within the Activation layer determines the presence of specific features at designated locations in the image. The Pooling layer selects the maximum values from the feature maps, which serve as inputs for the subsequent layer.

These initial layers are dedicated to feature extraction. Following these blocks, additional layers are incorporated into the network to execute the classification operation. Specifically, six more layers are introduced, including the Flattening layer, the Dropout layer, two fully-connected layers, another Activation layer employing the ReLU function, and the final Softmax layer.

The Flattening layer facilitates the conversion of a three-dimensional array into a vector. The output from the initial feature-extraction layers is in the form of a three-dimensional matrix. This layer transforms the input information into a vector format, enabling seamless handling by the subsequent layers.

The incorporation of a Dropout layer serves as a strategy to mitigate the challenge of network overfitting. Overfitting arises when the network adeptly classifies training patterns but struggles with novel patterns. Addressing overfitting can be approached through two avenues: expanding the size of the training set or diminishing the network’s complexity. In our specific case, where a predetermined set of images is available, the latter option is considered to alleviate overfitting. To this end, a Dropout layer is employed, which randomly deactivates a subset of neurons during training. This deactivation reduces the network’s reliance on the training set, thwarting the memorization of training data. By ensuring that not all neurons are active simultaneously, inactive neurons are precluded from learning during specific instances.

The Dense layer, constituting a fully-connected layer, facilitates the connection of every neuron in the preceding layer to each neuron in the subsequent layer. This layer bears resemblance to its counterpart in a MLP. Leveraging a fully-connected layer simplifies the learning of non-linear combinations of features derived from convolution operations. Although features obtained through convolutions are generally valuable for classification, their amalgamation through this layer often enhances their discriminative power. In summary, each block, comprising a Convolution layer, an Activation layer utilizing the ReLU function, and a Pooling layer, undertakes the extraction of salient features from images, introduces nonlinearity to the network, and diminishes the dimensionality of features, rendering them invariant to scale and translation. The initial layers within these blocks extract features from the images, while the subsequent layers culminate the classification operations. The augmentation of filters leads to the generation of additional feature maps, and an increase in convolutional layers results in the creation of progressively abstract feature maps as the CNN deepens.

The data used in this study are not subject to ethics committee regulations, as no sensitive data (relating to humans or animals) are handled, as described in Section 8.

## 3 Results

This section elucidates the procedural steps undertaken to formulate the requisite tests and derive the conclusive results presented in this article. The CNN was meticulously crafted utilizing the Keras framework, seamlessly integrated into the Tensorflow ecosystem. Leveraging the synergistic functionalities offered by both frameworks, the CNN design process was executed to harness their collective capabilities.

In order to execute computations in the cloud environment, a virtual machine instance has been instantiated on the Google Cloud^1^ platform, specifically within the SAAS services (Software as a Service) offered by Compute Engine^2^. The virtual machine is configured with the following specifications:

- Machine type: n1-standard-1
- CPU: 1 vCPU Intel Xeon 2.30GHz
- Architecture: x86_64
- L1d cache: 32 k
- L1i cache: 32 k
- L2 cache: 256 k
- L3 cache: 46080 K
- RAM: 3792 MB
- HDD: 30GB
- OS: Debian GNU/Linux 9.9 Stretch + TF 1–13

The comprehensive dataset for CNN application encompasses 94,080 images, with 840 images corresponding to each of the 112 initial pictograms. This dataset was utilized to establish the training, validation, and test sets for the network, with the selection of random images from the initial set. In this regard, and as a contribution to this work, the ORP-SIG-2024 dataset has been created (43). It consists of the original pictograms proposed in ISO 7010, along with a series of transformations aimed at enabling models that utilize it to achieve greater generalization capacity. This allows them to adapt to real-world environments where, in many cases, these pictograms are not as visible as they should be, or where they suffer from color loss, shape distortion, or other alterations. The dataset comprises 299 pictograms across five categories, each with a resolution of 800×800 pixels. There are 16 transformations for each pictogram, resulting in the following structure:

- E—Safety condition signals (68 originals and 1,020 modified).
- F—Fire equipment signals (19 originals and 304 modified).
- P—Prohibition signals (60 originals and 960 modified).
- M—Obligation signals (74 originals and 1,184 modified).
- W—Warning signals (78 originals and 1,248 modified).

The training and validation sets consist of 67,200 images distributed across 112 classes, resulting in 600 images per class. This dataset was randomly partitioned into two subsets, allocating 50,400 images for training (75%) and 16,800 images for validation (25%). The remaining 26,880 images constitute the test dataset, with 240 images allocated per class. The input images presented to the CNN are of dimensions 28 × 28 pixels to avoid computational resource saturation on the deployed virtual machine. As previously noted, the initial size reduction results in 100 × 100 pixels images, ensuring subsequent reductions do not lead to information loss within the image set.

Considering the paramount importance of parameter optimization during the training process, the definition of hyperparameters is a pivotal objective and challenge in implementing CNN-based techniques. Given the objective of this study and the acknowledgment that an optimal method for enhancing network performance does not exist, hyperparameters are considered as an integral part of the network structure. Several models, incorporating modifications based on (44) and its random search study, are proposed.

Before initiating the network training, it becomes imperative to define the number and size of filters. The utilization of more filters facilitates the extraction of additional image features, enhancing the network’s proficiency in pattern recognition. However, it is crucial to note that the number of filters also influences the training time required for the network.

Four distinct models have been conceptualized and scrutinized, adhering to the CNN architecture expounded. The primary aim is to assess the network’s performance under diverse configuration parameters. In the initial scenario, the focus is on scrutinizing the loss estimation (loss) and accuracy (accuracy) of the models. Subsequently, the comparative analysis delves into the models’ performance with test data that has not been previously exposed to the network.

The accuracy of a classification method is computed based on the number of correctly classified samples, CL, and the total number of test samples, TS, (3).


(3) $$\mathrm{accuracy}=\frac{CL}{TS}$$

The loss function, also called the cost function, measures the prediction error of the network. There are several functions that are commonly used as loss functions. In this case we have chosen the cross-entropy function (Equation 4), which is the most common choice for classification. In this equations M is the number of classes; log is the natural logarithm; y_o,c_ is the binary indicator (0 or 1) if class label c is the correct classification for observation o; p_o,c_ is the predicted probability observation o is of class c; A lower score indicates that the model is performing better. The training of all models was carried out by performing 8, 15, 20 and 30 iterations (epochs) on the training and validation sets.


(4) $$H\left(y,p\right)=-\overset{M}{\underset{c=1}{\sum }}y_{o,c}\log\left(p_{o,c}\right)$$

### 3.1 Model 1

Model 1 adheres to the CNN architecture illustrated in Figure 3. Table 1 provides a comprehensive breakdown of the architecture details when implemented through Keras and Tensorflow. In the table, the first column designates the name associated with each layer, while the second column denotes its type. All types of names correspond to those depicted in Figure 3, with the exception of Conv2D and MaxPool2D. Conv2D denotes a two-dimensional Convolution layer, and MaxPool2D signifies a Max-pooling layer designed for two-dimensional data. The third column of Table 1 elucidates the output shape of each layer. In all instances, the first argument signifies the batch size, and the value None accommodates variable batch size.

**Table 1:** Model 1: layers and characteristics.

Layer	Layer type	Output shape	Parameters
conv2d-1	Conv2D	(None, 28, 28, 32)	2,432
Activation-1	Activation	(None, 28, 28, 32)	0
max_pooling2d-1	MaxPooling2	(None, 14, 14, 32)	0
conv2d-2	Conv2D	(None, 14, 14, 64)	51,264
Activation-2	Activation	(None, 14, 14, 64)	0
max_pooling2d-2	MaxPooling2	(None, 7, 7, 64)	0
Flatten-1	Flatten	(None, 3,136)	0
Dropout-1	Dropout	(None, 3,136)	0
Dense-1	Dense	(None, 500)	1,568,500
Activation-3	Activation	(None, 500)	0
Dense-2	Dense	(None, 112)	56,112
Softmax-1	Softmax	(None, 112)	0

Subsequent layers process inputs and generate outputs of size 14 × 14. The second Max-pooling layer further reduces the input from 14 × 14 to 7 × 7. The Flattening layer produces a vector with 7 × 7 × 64 = 3,136 elements. The two fully connected Dense hidden layers subsequently decrease the output size (with 500 outputs for the first and only 112 for the second). Finally, the Softmax layer culminates in 112 outputs, corresponding to the diverse pictograms the network aims to identify. The last column in Table 1 delineates the number of parameters (weights) within each layer, where a value of 0 implies the layer does not undergo any learning.

As previously outlined, two groups of three layers (Convolution, Activation with ReLU, and Max-pooling) were employed. In this instance, the first group assimilates knowledge from 32 filters, while the second group incorporates 64 filters. Results pertaining to the various convolutions are presented in Table 2, with a training time of 50 min. Notably, an increase in the number of iterations correlates with a more refined learning process, leading to improved precision in pictogram classification by the network.

**Table 2:** Results of Model 1.

	Training		Validation	
	Loss	Accuracy	Loss	Accuracy
8 epochs	0.8972	0.7308	0.8316	0.7583
15 epochs	0.4237	0.8748	0.3429	0.9064
20 epochs	0.5951	0.8872	0.5518	0.8911
30 epochs	0.1630	0.9530	0.2108	0.9581

### 3.2 Model 2

Model 2 retains the identical layers as Model 1; nevertheless, there is a modification in the number of filters within the two initial groups. Specifically, Model 2 employs 20 filters in the first group and 50 filters in the second. This adjustment aims to investigate the impact of reducing parameters on the network’s outcomes. A comprehensive breakdown of the Model 2 architecture is presented in Table 3.

**Table 3:** Model 2: layers and characteristics.

Layer	Layer type	Output shape	Parameters
conv2d-1	Conv2D	(None, 28, 28, 20)	1,520
Activation-1	Activation	(None, 28, 28, 20)	0
max_pooling2d-1	MaxPooling2	(None, 14, 14, 20)	0
conv2d-2	Conv2D	(None, 14, 14, 50)	25,050
Activation-2	Activation	(None, 14, 14, 50)	0
max_pooling2d-2	MaxPooling2	(None, 7, 7, 50)	0
Flatten-1	Flatten	(None, 2,450)	0
Dropout-1	Dropout	(None, 2,450)	0
Dense-1	Dense	(None, 500)	1,225,500
Activation-3	Activation	(None, 500)	0
Dense-2	Dense	(None, 112)	56,112
Softmax-1	Softmax	(None, 112)	0

The outcomes derived from this model are delineated in Table 4. Comparative analysis between Model 1 and Model 2 reveals that the disparities are not markedly substantial. Despite the fact that Model 2 utilizes less training time, 44 min, it yields inferior results compared to Model 1. Remarkably, the reduction in the number of filters within the convolution layers in Model 2 does not significantly impact the overall results.

**Table 4:** Results of Model 2.

	Training		Validation	
	Loss	Accuracy	Loss	Accuracy
8 epochs	0.9105	0.7124	0.8607	0.7457
15 epochs	0.4503	0.8583	0.3719	0.8917
20 epochs	0.6197	0.8681	0.5774	0.8791
30 epochs	0.1798	0.9391	0.2402	0.9472

### 3.3 Model 3

Model 3 builds upon the framework established by Model 2, aiming to attain an elevated level of abstraction. The novel model incorporates an additional set of layers, specifically Convolution, Activation, and Max-pooling, mirroring the structure of the initial two groups present in Model 2. Notably, the first two groups of layers maintain an identical number of filters as employed in Model 2, while the third group distinguishes itself by utilizing 50 filters. Table 5 delineates the architecture implemented for Model 3.

**Table 5:** Model 3: layers and characteristics.

Layer	Layer type	Output shape	Parameters
conv2d-1	Conv2D	(None, 28, 28, 20)	1,520
Activation-1	Activation	(None, 28, 28, 20)	0
max_pooling2d-1	MaxPooling2	(None, 14, 14, 20)	0
conv2d-2	Conv2D	(None, 14, 14, 50)	25,050
Activation-2	Activation	(None, 14, 14, 50)	0
max_pooling2d-2	MaxPooling2	(None, 7, 7, 50)	0
conv2d-3	Conv2D	(None, 7, 7, 50)	62,550
Activation-3	Activation	(None, 7, 7, 50)	0
max_pooling2d-3	MaxPooling2	(None, 3, 3, 50)	0
Flatten-1	Flatten	(None, 450)	0
Dropout-1	Dropout	(None, 450)	0
Dense-1	Dense	(None, 500)	225,500
Activation-4	Activation	(None, 500)	0
Dense-2	Dense	(None, 112)	56,112
Softmax-1	Softmax	(None, 112)	0

As illustrated in Table 6, the outcomes yielded by this model exhibit inferior performance compared to its predecessors with a training time of 48 min. This observation suggests that, in our context, the introduction of an additional level of abstraction has not led to an enhancement in CNN results. Furthermore, a noteworthy decline in learning levels is evident in comparison to Models 1 and 2. This decline may be attributed to the diminishing input size of the image after multiple convolutional operations, reaching a point where further extraction of differentiable features becomes impractical.

**Table 6:** Results of Model 3.

	Training		Validation	
	Loss	Accuracy	Loss	Accuracy
8 epochs	0.9601	0.7005	0.8789	0.7313
15 epochs	0.4887	0.8510	0.3861	0.8842
20 epochs	0.6711	0.8621	0.5995	0.8694
30 epochs	0.2347	0.9237	0.2532	0.9341

### 3.4 Model 4

The final model under consideration bears resemblance to Model 1, with a notable modification involving the augmentation of filters in the initial convolutional layer, which is in close proximity to the input image. Precisely, 64 filters have been incorporated, doubling the count employed in Model 1. The architectural details of Model 4 are elucidated in Table 7. The outcomes presented in Table 8 closely resemble those derived from Models 2 and 3, with a training time of 53 min. This suggests that the augmentation of filters in the initial convolutional layer does not yield improvement in pictogram recognition.

**Table 7:** Model 4: layers and characteristics.

Layer	Layer type	Output shape	Parameters
conv2d-1	Conv2D	(None, 28, 28, 64)	4,864
Activation-1	Activation	(None, 28, 28, 64)	0
max_pooling2d-1	MaxPooling2	(None, 14, 14, 64)	0
conv2d-2	Conv2D	(None, 14, 14, 64)	102,464
Activation-2	Activation	(None, 14, 14, 64)	0
max_pooling2d-2	MaxPooling2	(None, 7, 7, 64)	0
Flatten-1	Flatten	(None, 3,136)	0
Dropout-1	Dropout	(None, 3,136)	0
Dense-1	Dense	(None, 500)	1,568,500
Activation-3	Activation	(None, 500)	0
Dense-2	Dense	(None, 112)	56,112
Softmax-1	Softmax	(None, 112)	0

**Table 8:** Results for Model 4.

	Training		Validation	
	Loss	Accuracy	Loss	Accuracy
8 epochs	0.9421	0.7309	0.9392	0.7391
15 epochs	0.4679	0.8753	0.4494	0.8897
20 epochs	0.6451	0.8878	0.6603	0.8746
30 epochs	0.2113	0.9498	0.3151	0.9387

## 4 Discussion and conclusion

**Figure 4:**
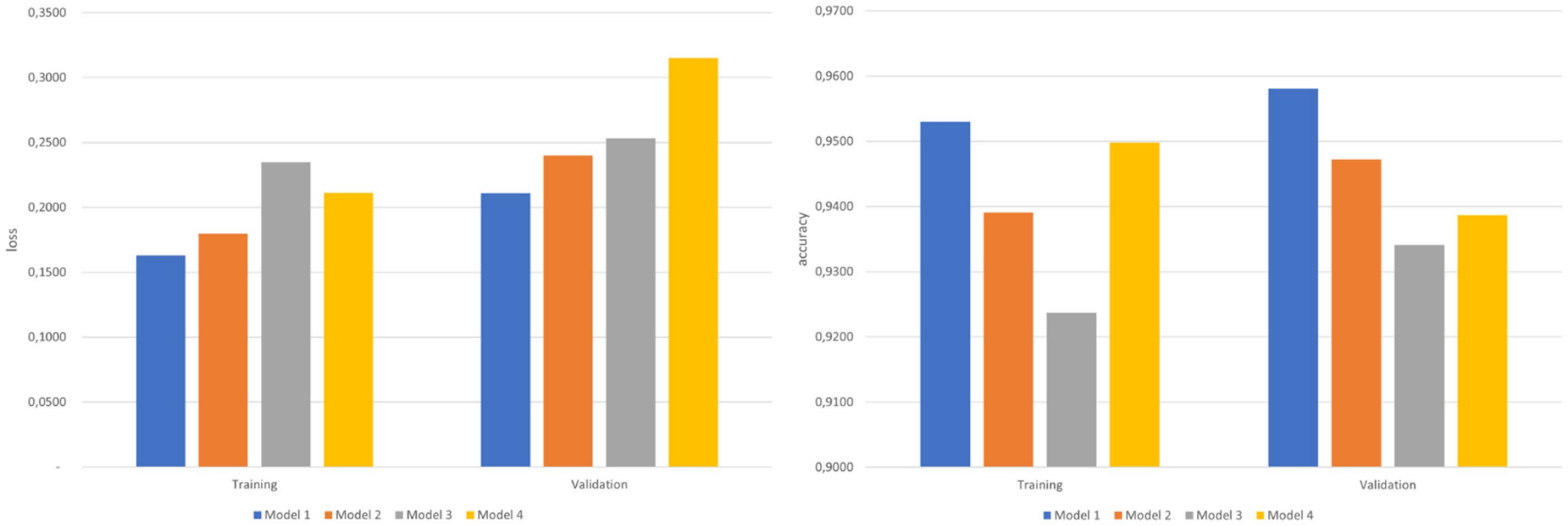
Loss and accuracy of the models for the training and the validation sets after 30 epochs.

Following the execution of experimentation with the four proposed architectures, Figure 4 depicts the optimal outcomes achieved by each model after 30 epochs. It is evident that the architecture corresponding to Model 1 stands out as the most successful. This model exhibits the highest accuracy for both the validation set (95.81%) and the test set (95.30%). Additionally, it attains the lowest loss values for both sets of images.

In contrast, Model 3 demonstrates the least favorable overall performance, signaling that the introduction of more layers to achieve a heightened level of abstraction does not contribute to the improvement of the CNN. Models 2 and 4, distinguished by the number of filters employed, both yield results inferior to Model 1. However, upon comparing these variants, it becomes apparent that the model utilizing a greater number of filters outperforms the one with fewer filters in a global context.

A thorough examination of the classification results has revealed that errors in classification predominantly occur among pictograms within the same group. To clarify, a pictogram featuring a prohibition sign might be mistakenly identified as another pictogram from the same group. It is noteworthy that despite this type of error, the positive aspect lies in accurately determining the group of signs to which the pictogram belongs. This precision allows for the notification of the general type of risk to the worker.

The use of metrics, such as loss and accuracy, in machine learning models, particularly in neural networks, is justified as each evaluates complementary aspects of performance. Loss measures how well the model minimizes errors between predictions and actual values, providing a continuous signal to optimize the model. Accuracy, on the other hand, measures the percentage of correct predictions, making it useful for assessing the overall effectiveness of the model in classification tasks. Using both metrics ensures a comprehensive evaluation of the model, optimizing both training and practical performance.

Considering the challenges posed by the inclusion of a substantial number of distorted pictograms in the training set, the CNN’s classification results can be deemed satisfactory. To further evaluate the model’s efficacy in scenarios involving undistorted images, a new test was conducted using only the original, non-transformed pictograms. This set of images, not utilized in the training or validation phases, offers insights into the model’s performance in real-world scenarios where images remain unaltered.

**Figure 5:**
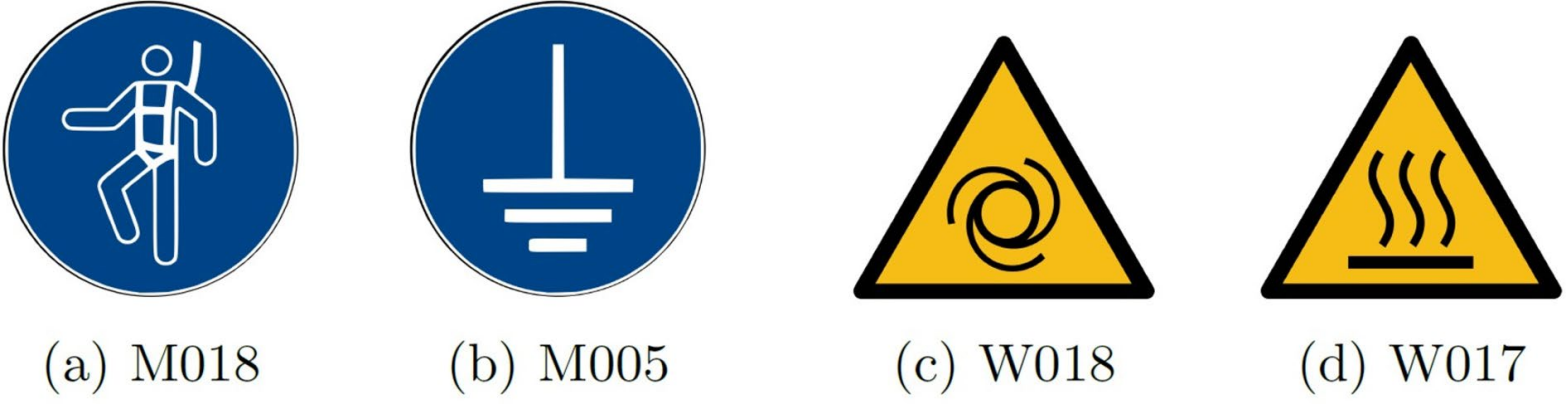
Original pictograms not correctly identified by the model. When pictogram (a) was presented to the CNN, the network identified pictogram (b). The same happened for pictograms (c,d).

For this evaluation, the architecture proposed in Model 1, which demonstrated superior overall results, was employed. The results of this test were highly promising, with the proposed model achieving nearly perfect classification, accurately identifying 110 out of 112 images. Figure 5 illustrates the misclassified pictograms, highlighting that in both cases, the erroneously identified pictogram belongs to the same group as the intended one. This observation suggests that, considering the size of the pictograms and the contrast and position of colors within them, these failures are acceptable. The overall accuracy for the original pictograms reaches an impressive 99.98%.

The presented CNN has been applied to images depicting Occupational Safety and Health Administration safety signs, encompassing various simulated contexts to emulate potential deterioration in real-world scenarios. Computational results demonstrate the viability of the models in effectively classifying signs within these images. It is crucial to emphasize that apart from the simulated deterioration, signs may also be subject to breakage or partial loss, resulting in incomplete pictograms. While signs exhibiting such issues should ideally be replaced, various tests were conducted with these types of signals to comprehensively analyze the proposed methods in this article.

**Figure 6:**
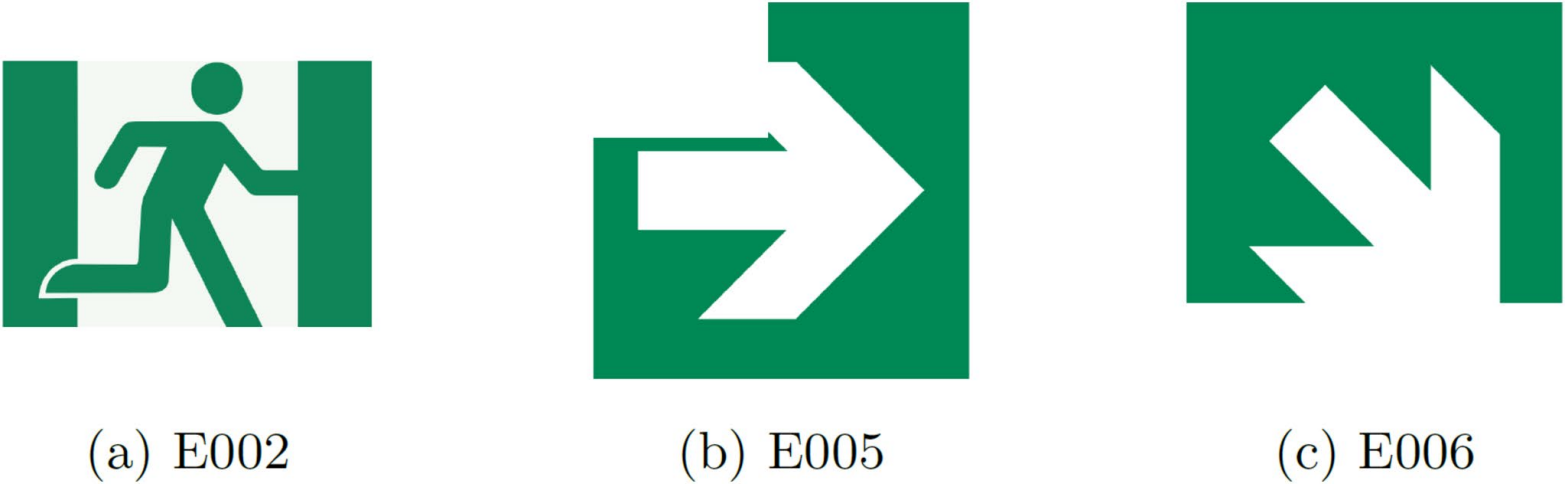
Pictograms with missing parts.

These tests serve the dual purpose of assessing the generalization capability and accuracy of the sign classification process. To this end, safety signs depicted in Figure 6 were employed.

Given that Model 1 demonstrated superior performance after 30 epochs, this configuration was employed in the truncated pictogram tests. As illustrated in Table 9, the classification of the examined truncated pictograms is highly accurate. The success rate for pictogram E002 is 97.65%, while for pictograms E005 and E006, it reaches 100%, effectively generalizing from the original pictograms. It is crucial to note that the truncation of these pictograms is partial, eliminating a portion of the pictogram. It is evident that if the truncation were to impact a significantly larger segment of the pictogram, image recognition would pose a formidable challenge even for a skilled human observer.

**Table 9:** Results of Model 1: pictograms with missing parts.

Pictogram	Prediction	Accuracy
E002 truncated	E002	0.9765
E005 truncated	E005	1
E006 truncated	E006	1

The exploration of truncated pictograms presents an intriguing avenue for future research. Multiple sets of pictograms featuring varying degrees of deterioration could be examined to assess the network’s performance. In such cases, determining the level of deterioration at which the network’s response remains acceptable would be of interest. Clearly, to enable the CNN to generate accurate results for truncated images missing substantial portions, it would be advisable to include such images in the training set of the network.

Considering that there are no works related to signal recognition in the field of ORP, the results obtained in different works on traffic signal recognition are presented for comparison. Although it is true that they do not correspond to the same type of pictograms, the results can be compared because the shape of the signals and their colors, in most cases, are the same.

In the research presented in (45), the presented system is based on a Mask R-CNN architecture where, unlike traditional approaches with characteristics, it is applied to a wide range of categories, where individual instances of traffic signals are not only subject to changes in lighting conditions, scale, viewing angle, blur, and occlusions but also to significant appearance variations. In this case, the authors achieve an accuracy of 97.5% in evaluating the proposed model.

In this work (38), the evaluation of signal recognition is carried out with YOLOv5 (46) and SSD (47). The results obtained in this study reach an average accuracy of 97.6% for the different types of signals, and in the case of SSD, it reaches an average of 90.1%. Considering the results obtained from various studies and datasets that do not correspond exactly to the pictograms proposed in our work but are comparable, the model presented in this research achieves accuracy rates exceeding 95%, which aligns with previous findings. The focus of this study has been on assessing the feasibility of a Deep Learning-based model capable of accurately recognizing ORP signals. This model aims to be implemented in workplaces to enhance worker safety significantly.

Simultaneously, the creation of the specific ORP-SIG-2024 dataset has been undertaken to facilitate experimentation in this domain. This dataset allows for the exploration of various technologies aimed at improving worker safety. The overarching goal is to enhance worker safety through the analysis of different approaches and technologies applied to this dataset.

ORP holds paramount significance for workers across diverse fields. A workplace’s optimal performance and the judicious use of necessary materials are crucial for averting accidents that pose substantial costs to both workers and companies. Safety signs, integral to ORP, serve to alert workers to potential dangers in specific work environments. However, negligence or overconfidence at times hampers the requisite attention, and the correct visualization of signs is impeded by factors such as their location or deterioration.

This article proposes the utilization of a CNN to automatically classify images related to ORP. The network processes an input image containing a safety sign, which may be damaged or distorted, and identifies the safety sign. Employing a CNN for this purpose necessitates a sufficiently large dataset for training. Given the absence of public datasets for ORP safety signs, the research described herein involved the generation of an extensive image set using a method that starts with ISO 7010 compliant pictograms. This initial set was expanded by applying transformations simulating image degradation and varying perspectives. Computational results affirm that the CNN trained with the augmented image set exhibits commendable classification capabilities.

Four CNN models were tested to investigate the impact of the number of layers and filters. Results analysis reveals that all models yield effective solutions with comparable training times, but Model 1 outperforms the others, achieving an accuracy exceeding 95%.

The examination of different CNN configurations indicates that augmenting layers and parameters does not necessarily improve results. Successful model development relies heavily on experiential knowledge in determining network architecture and parameter estimation. The success of the experiment, surpassing expectations, underscores the efficacy of artificially creating the dataset. Training the CNN with the transformed image set enhances the network’s generalization capacity significantly.

**Figure 7:**
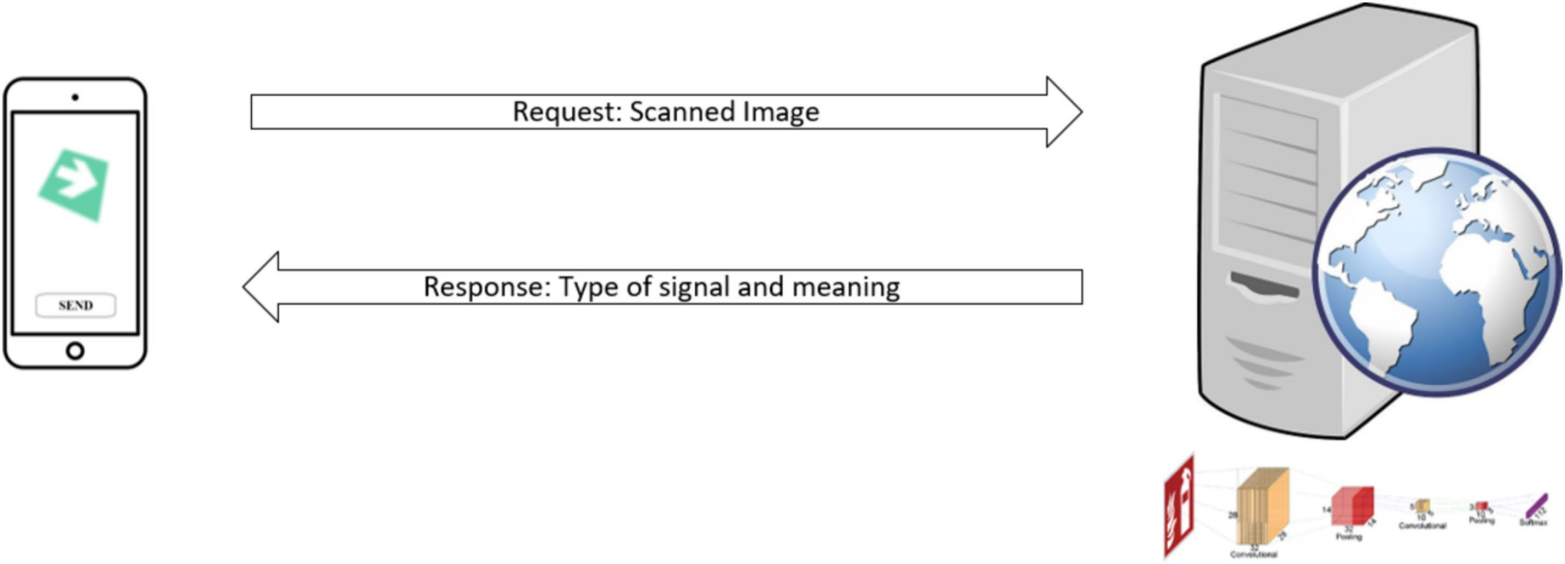
Architecture deployment scheme for the implementation of the model.

The next logical step involves implementing systems that rigorously evaluate both the effectiveness and practical utility of the proposed model. This process is crucial to ensure that the model not only works properly in controlled environments but is also robust and effective in real-world scenarios, where variability in conditions can be greater. To achieve this goal, the overall strategy is based on automating the process using devices that operate within a service-oriented architecture, as is represented in Figure 7. This approach allows for greater flexibility and scalability by separating data capture from computationally intensive processing.

Specifically, image capture is performed using a mobile device, which serves as a lightweight and portable interface for the user, facilitating real-time data collection. This choice enhances the system’s accessibility in various work environments. Subsequently, the captured images are sent to an application server, where the convolutional model is executed. Given that the processing of CNNs requires considerable computational capacity, the server handles this task to relieve the load on mobile devices and ensure a rapid and efficient response.

Once the server has processed the image and applied the CNN model to classify the safety sign, a response is generated that includes both a brief identification of the signal and a more detailed description. The latter is particularly useful for users with less experience in identifying safety signs, as it provides additional information about the risks associated with the recognized signal.

The server can be deployed in either a local or a remote network, offering flexibility depending on the specific application requirements. The decision between local or remote deployment will depend on various factors, such as communication latency and the need for real-time processing. In critical applications where response time is essential, using a local network could reduce delays, while a remote server may be more suitable for scenarios requiring greater processing capacity or distributed access.

Additionally, the system can be optimized by integrating real-time cameras into personal protective devices, such as safety helmets. These cameras would continuously capture images and send the information to the server for analysis and classification, thereby enhancing worker safety through automated detection and response to warning signals in the workplace. This integration strengthens the system’s ability to operate in dynamic conditions, providing active and adaptive protection in real-time.

## Data Availability

The datasets presented in this study can be found in online repositories. The names of the repository/repositories and accession number(s) can be found below: https://data.mendeley.com/datasets/dfg5hnxrzg/1.

## Footnotes

^1^ https://cloud.google.com/gcp/

^2^ https://cloud.google.com/compute/
