# Supplementary material for: Machine vision-based recognition of safety signs in work environments
Source: Front Public Health. 2024 Nov 27;12:1431757. doi: 10.3389/fpubh.2024.1431757 (PMC11631886; doi:10.3389/fpubh.2024.1431757)
Supplement: Supplementary file 1 [file Table_1.DOCX]

Supplementary Material

# Algorithms

This supplementary material includes the algorithms that have been used to apply transformations to the images and expand the set available to train the CNN. The variables used as parameters of the algorithms are defined as follows. In this way, *imgsRoute* is the path of the image*; pathNewSet* is the path where the generated images will be deployed*; nameNew* is the path and leading part of the name of a new image; *listPictogram* is the list of images generated when applying a transformation; *listPictogramAux* is the list of images to which the transformation will be applied; *i* is the counter of total images generated so far; *color* is the background color; *rotated* is the input image to apply the transformation described the Algorithms.

**Algorithm 1** Transformations applied to generate the set of images to be used by the CNN

**Require:** *imgsRoute, pathNewSet, angFrom, angTo, angStep, color*

1: **for each** route **in** imgsRoute **do**

2: *nameNew ← pathNewSet* + */imgName*(*route*)*/*

3: *i ←* 0

4: **for each** ang **in** list(angFrom,angTo,angStep) **do**

5: *listPictogram ← listEmpty*

6: *image ← removeTransparency*(*image, color*)

7: *rotated ← scale*(*rotate*(*image, ang,* 100))

8: *listPictogram ← listPictogram* + *rotated*

9: *saveImage*(*nameNew* + *i* + *R.png, rotated* )

10: *i ← i* + 1

11: PERSPECTIVE-TR (*nameNew*, *listPictogram*, *rotated* , *i*, *color*)

12: *listPictogramAux ← listPictogram*

13: REDUCTION-REPOSITIONING-TR

(*nameNew*, *listPictogram*, *listPictogramAux*, *i*, *color*)

14: *listPictogramAux ← listPictogram*

15: CONTRAST-BRIGHTNESS-TR (*nameNew*, *listPictogram*, *listPictogramAux*, *i*)

16: *listPictogramAux ← listPictogram*

17: BLUR-TR (*nameNew*, *listPictogram*, *listPictogramAux*, *i*)

18: **end for**

19: **end for**

**Algorithm 2** PERSPECTIVE-TR

**Require:** *newName, listPictogram, rotated, color*

1: *listPictogram ← listEmpty*

2: **for each** num **in** list(1,4) **do**

3: *imgP* = *perspective*(*rotated, orientation, random*(1*px,* 30*px*)*, color*)

4: *saveImage*(*nameNew* + *i* + *P.png, imgP*)

5: *i ← i* + 1

6: *listPictogram ← listPictogram* + *imgP*

7: **end for**

**Algorithm 3** REDUCTION-REPOSITIONING-TR

**Require:** *newName, listPictogram, listPictogramAux, i, color*

1: *listPictogram ← listPictogramAux*

2: **for each** img **in** list(1,4) **do**

3: *imR ← fitReduce*(*img, color*)

4: *saveImage*(*nameNew* + *i* + *RD.png, imgR*)

5: *i ← i* + 1

6: *listPictogram ← listPictogram* + *imgR*

7: **end for**

**Algorithm 4** CONTRAST-BRIGHTNESS-TR

**Require:** *newName, listPictogram, listPictogramAux, i*

1: *listPictogram ← listEmpty*

2: **for each** img **in** listPictogramAux **do**

3: *imgCB ← contrastBrightness(img, random())*

4: *saveImage(nameNew + i + CB.png, imgCB)*

5: *i ← i* + 1

6: *listPictogram ← listPictogram* + *imgCB*

7: **end for**

**Algorithm 5** BLUR

**Require:** *newName, listPictogram, listPictogramAux, i*

1: *listPictogram ← listEmpty*

2: **for each** img **in** listPictogramAux **do**

3: *imgB ← blur(img, random())*

4: *saveImage(nameNew + i + B.png, imgB)*

5: *i ← i* + 1

6: *listPictogram ← listPictogram* + *imgB*

7: **end for**
